# Genome-Wide Prediction of Functional Gene-Gene Interactions Inferred from Patterns of Genetic Differentiation in Mice and Men

**DOI:** 10.1371/journal.pone.0001593

**Published:** 2008-02-13

**Authors:** Zoltán Bochdanovits, David Sondervan, Sophie Perillous, Toos van Beijsterveldt, Dorret Boomsma, Peter Heutink

**Affiliations:** 1 Section Medical Genomics, Department of Clinical Genetics, Vrije Universiteit Medisch Centrum (VUMC), Amsterdam, The Netherlands; 2 Center for Neurogenomics and Cognitive Research, Vrije Universiteit (VU), Vrije Universiteit Medisch Centrum (VUMC), Amsterdam, The Netherlands; 3 Department of Biological Psychology, Vrije Universiteit (VU), Amsterdam, The Netherlands; Washington University in St. Louis School of Medicine, United States of America

## Abstract

The human genome encodes a limited number of genes yet contributes to individual differences in a vast array of heritable traits. A possible explanation for the capacity our genome to generate this virtually unlimited range of phenotypic variation in complex traits is to assume functional interactions between genes. Therefore we searched two mammalian genomes to identify potential epistatic interactions by looking for co-adapted genes marked by excess two-locus genetic differentiation between populations/lineages using publicly available SNP genotype data. The practical motivation for this effort is to reduce the number of pair-wise tests that need to be performed in genome-wide association studies aimed at detecting G×G interactions, by focusing on pairs predicted to be more likely to jointly affect variation in complex traits. Hence, this approach generates a list of candidate interactions that can be empirically tested. In both the mouse and human data we observed two-locus genetic differentiation in excess of what can be expected from chance alone based on simulations. In an attempt to validate our hypothesis that pairs of genes showing excess genetic divergence represent potential functional interactions, we selected a small set of gene combinations postulated to be interacting based on our analyses and looked for a combined effect of the selected genes on variation in complex traits in both mice and man. In both cases the individual effect of the genes were not significant, instead we observed marginally significant interaction effects. These results show that genome wide searches for gene-gene interactions based on population genetic data are feasible and can generate interesting candidate gene pairs to be further tested for their contribution to phenotypic variation in complex traits.

## Introduction

The presence of epistatic interactions between genes has fundamental consequences for the course and outcome of evolution by natural selection [1]–[4] and results in the emergence of co-adapted gene complexes, i.e. combinations of variants at different genes that give a selective advantage only when both are present in the same individual. Consequently, it should be possible to detect genes involved in epistatic interactions from a population genetic approach, without prior knowledge of the specific phenotypes that are affected. Following up on the original concept introduced by Sewall Wright [1], [2] we searched for co-adapted gene complexes by looking for excess genetic divergence between populations/lineages at two-locus genotypes. The basic rationale is that if a specific combination of alleles at different genes outperforms the alternative combination(s), than not all two-locus genotypes will be observed in their expected frequencies. Within a population selection for a trait underlined by epistatic gene effects will maintain linkage disequilibrium between the loci involved [5], because specific allelic combinations will be systematically removed. Between (sub-) populations the same process will generate genetic differentiation because different combinations of alleles may perform equally well, resulting in phenotypically equivalent but genetically different populations [2]. This theoretical prediction of Wright's Shifting Balance Theory of evolution has empirical support [4]. In essence, by looking for deviations from equilibrium two-locus genotype frequencies, we aim to detect the signature of natural selection [6]–[9] on pairs of genes from population genomic data.

We explored two mammalian genomes for functional gene-gene interactions and followed up a selection of candidate gene pairs to detect a possible joint effect on phenotypic variation in a complex trait in both mice using a well characterized set of mouse lines (BxD) and in humans using a Dutch twin cohort.

Recombinant inbred lines (RILs) are generated by inbreeding the progeny of two parental lines that are themselves inbred. As a result an individual line can have only one of a possible four two-locus genotypes: aabb, aaBB, AAbb or AABB and all are expected to occur in equal frequencies in a large collection of RILs. However, if these four genotypes do not equally persist through the many generations of inbreeding, i.e. if there is a selective advantage of a specific combination of alleles, than not all two-locus genotypes will be observed in equal frequencies. In other words, in a large set of RILs we might observe significant Linkage Disequilibrium (LD) between physically unlinked genes if these genes jointly affect an adaptive phenotype [5] and some combinations are more likely than others to survive the process of inbreeding. In fact, this idea has been pursued before in the context of signatures of reproductive isolation and shown to reveal patterns consistent with epistatic gene interactions that arise in the shape of Dobzhansky-Muller incompatibilities [10], [11].

In contrast to the mouse data, the available human genotypes were derived from outbred, ethnically distinct populations. In this case pairs of functionally interacting genes can be detected following a slightly different approach. Considering only bi-allelic loci such as SNPs, two-locus genotypes are transmitted from one generation to another in one out of four possible combinations: for the AaBb genotype the gamets can be Ab, AB, ab, aB. If selection favors one of these configurations (i.e. virtual “haplotypes” or “trans-haplotypes” when the loci are unlinked) above the others, than excess genetic differentiation between three major human populations at the level of trans-haplotype frequencies may indicate epistatic interaction between genes for the reasons outlined above. In contrast to the recombinant inbred lines, where phase is evident, in outbred human populations trans-haplotype frequencies had to be estimated. From these frequencies F_st_ was calculated which is a measure of genetic distance between populations originally introduced by Sewall Wright. When considering natural populations a known complication of this approach is the confounding effects of selection and demography [12], i.e. a large genetic distance alone need not be sufficient evidence for natural selection. To allow for straightforward interpretation of our results and in addition to conducting coalescent simulations we restricted ourselves to a comparison of non-synonymous coding SNPs (NSCS) vs. neutral SNPs (NS). NSCS change the amino acid sequence of proteins and are therefore natural candidates to be involved in functional interactions, in contrast to combinations of NS that will provide an estimate of the background levels of genetic differences between populations. Because neutral, demographic processes have the same effect on all SNPs, any difference we might observe between the two classes, after correcting for the difference in their allele frequency spectra, should reflect the functional properties of NSCS.

## Results

### LD between unlinked loci in the mouse genome

**Figure 1:**
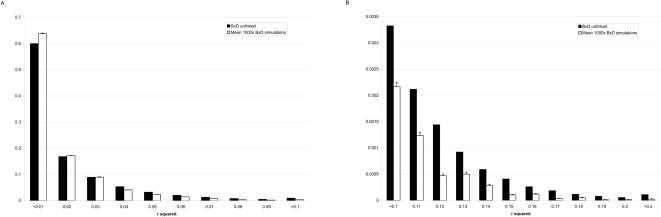
Frequency distribution of LD between unlinked SNPs in the BxD mouse recombinant inbred lines. A Black bars represent the observed frequency of pairs of unlinked SNPs at increasing levels of LD (measured as r^2^). White bars show the distribution of LD in 1000 simulated sets of RILs. The errors bars are standard deviations. As expected the distribution of the observed data is shifted to the right. B In the right tail of the distribution, the excess of unlinked SNPs that exhibit very strong LD relative to the neutral expectation remains consistently significant. It should be noted that very high levels of LD were observed in the simulated dataset but these are clearly outnumbered by the real data.

Based on publicly available genotype data on 89 BxD mouse RILs (http://www.genenetwork.org/dbdoc/BXDGeno.html) we calculated LD and corresponding p-values for 6,659,288 pairs of physically unlinked, autosomal SNPs (Table S1 and S2). Unlinked polymorphisms are not expected to be in LD, yet we observe very strong correlations between SNPs on different chromosomes. The lowest p-value in this dataset was 1.5×10^−12^, in fact 707 combinations of unlinked SNPs were in significant LD even after Bonferroni correction for 6,659,288 tests. As RILs are derived from a known breeding scheme, any significant result should not be caused by demography. Nevertheless it is straightforward to simulate RIL genotypes and determine empirically the expected distribution of LD between unlinked loci. We generated 1000 replicates of 89 RILs with 3800 SNPs equally distributed across the genome. The simulations showed that very high levels of LD may be present in RILs just by chance, however, starting from r^2^>0.04, the real data consistently exhibits a significant excess of non-independent combinations (Figure 1), when compared to the average and standard deviation observed in the 1000 replicate simulations. These results clearly show that a large number of unlinked polymorphisms are co-occurring non-independently in the mouse genome. We propose that the most likely explanation of the data is that the specific combinations of SNPs that appear to be in strong LD in fact identify functionally interacting genes that have conveyed a selectively advantage during the process of inbreeding. The genome-wide prevalence of such epistatic interactions can be roughly estimated by quantifying the excess frequency of “real” interactions at those (higher) levels of LD where observed data is consistently in the majority. Several percent of all pairs (Figure 1) are implicated suggesting that the total number of functional interactions is at least a couple of times larger than the number of genes in the mouse genome.

### Gene-gene interactions in phase II detoxification determine alcohol preference in mice

Given our interpretation that pairs of unlinked polymorphisms in strong LD represent functional interactions, we aimed to establish the extent to which the interaction between genes rather than their individual effect influences phenotypic variation in complex traits. For this purpose we selected a small subset of SNP pairs in strong LD. To enhance our chances to observe a real and sizeable gene-gene interaction effect we focused on the set of 707 combinations of SNPs that were in significant LD even after Bonferroni correction. In addition, we considered only gene related SNPs, for our purposes defined as either in coding sequence, splice site or mRNA 5′ and 3′ UTRs, because these directly implicate a specific gene. From the 707 SNP pairs we selected those 12 combinations where both SNPs marked a specific gene (Table 1). The purpose of this selection was to facilitate the interpretation of any association we might find.

**Table 1:** 15 unlinked gene based SNPs involved 12 pair-wise combinations that exhibit significant LD after Bonferroni correction.

Chr SNP1	Chr SNP2	SNP1	SNP2	Gene 1	Gene 2	LD p-value
2	6	rs13459064	rs13478608	Nrf2	Pon2	4.21×10^−09^
2	6	rs13459064	rs6343757	Nrf2	Dync1i1	4.21×10^−09^
2	6	rs13459064	rs13478618	Nrf2	Glcci1	1.20×10^−11^
3	7	rs13459183	rs13479546	LOC665113	Ptpre	5.3×10^−11^
3	7	rs6260196	rs13479546	Slc2a2	Ptpre	7.59×10^−10^
5	10	rs6263715	rs13480653	1810013D10Rik	Rtdr1	3.44×10^−09^
7	14	rs13479126	rs13482205	V1rg3	Rcbtb1	6.22×10^−10^
7	14	rs13479126	rs3710549	V1rg4	Sacs	2.86×10^−09^
6	6	rs13478608	rs3682699	Pon2	Lmo3	4.66×10^−09^
6	6	rs6343757	rs3682699	Dync1i1	Lmo3	4.66×10^−09^
6	6	rs13478618	rs3682699	Glcci1	Lmo3	7.11×10^−09^
8	8	rs13459102	rs13479838	Tm2d2	9130012O13Rik	1.51×10^−12^

The SNPs present on the same chromosome are more than 50 cM apart.

**Figure 2:**
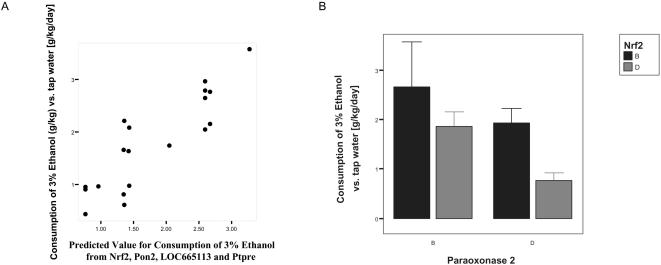
Consumption of 3% ethanol explained by the interaction between genes involved in oxidative stress and detoxification. A Correspondence between the observed amount of ethanol consumed by BxD mice and the predicted values based on the genotype at four genes. The correlation between the observed and predicted values is 81% and the pattern is significant after Bonferroni correction for testing 626 phenotypes at p = 6.5×10^−5^. B Ethanol consumption of the four two-locus genotype classes based on Paraoxonase 2 and Nrf2. The error bars are standard error of the mean. The interaction between the two genes is apparent as the effect size of the increaser genotype at either gene depends on the genotype at the other locus. This pattern is very likely to be biologically relevant as Nrf2 is a known regulator of the expression of antioxidant proteins and Paraoxonase 2 is an antioxidant protein.

We used these 12 combinations of 15 gene based SNPs as predicting variables on the 626 phenotypes publicly available for a subset of the BxD RILs. To avoid fitting an over-parameterized model, in the initial screen only the interaction terms were included. The significance of the full model was evaluated compared to a model including only the intercept. We found one phenotype to be significantly associated with the genotype data, corrected for the number of tests performed. Following up this one phenotype and after excluding those variables that were not contributing to the prediction we found that 2 combinations of 4 different gene-based SNPs explained 66 % (adjusted r^2^) of the phenotypic variation (p = 6.5×10^−5^) in the amount of 3% ethanol consumed per 24 hours when given ad libitum access (Figure 2). When introduced to the model none of the four main effects were significant, in agreement with our expectation that the interaction terms would be of relevance, although admittedly the significance level of the two interaction terms were reduced to p = 0.04 and a marginally non-significant p = 0.06.

The four genes implicated in ethanol preference in mice are: Nrf2 (rs13459064) interacting with Paraoxonase 2 (rs13478608) and LOC665113 (similar to Traf2 and NCK-interacting kinase) (rs13459183) interacting with Ptpre (rs13479546). The biological functions of the latter two are not yet known. In contrast, paraoxonase 2 is an antioxidant protein and Nrf2 is a known regulator of gene expression of antioxidant proteins and phase II detoxification enzymes [13]. Although the interaction between paraoxonase 2 and Nrf2 is marginally non-significant, the possible involvement of Nrf2 alcohol preference in mice is remarkable, as it has been recently shown that a promoter polymorphism in the phase II detoxification enzyme NQO2 plays an important role in the pathogenesis of alcoholism and alcohol withdrawal symptoms in humans [14]. Nrf2 is known to directly regulate the expression of NQO2 [15] meaning that our tentative finding is plausible based on the known biological function of the genes involved. Clearly, more experimental studies would be necessary to establish the link between paraoxonase 2 and Nrf2 and ethanol consumption, but the present results are consistent with the prediction that unlinked loci in strong LD might be functionally interacting.

### Genetic differentiation of two-locus genotypes between three human populations

**Figure 3:**
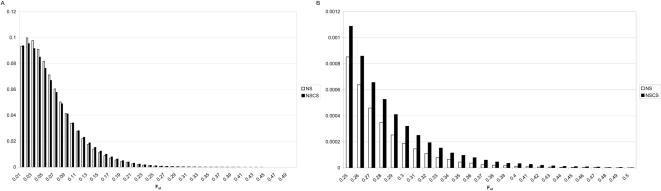
Frequency distribution of genetic distance between three major human populations at pairwise combinations of non-synonymous coding vs. neutral SNPs. A Black bars represent the observed frequency of F_st_ calculated for combinations of NSCS at different genes. A higher value represents a larger genetic distance between populations considering two genes at a time. White bars represent the observed frequency calculated from randomly selected NS. The distribution based on NSCS is shifted to the right. B Starting from relatively low levels of genetic differentiation the NSCS derived trans-haplotypes are significantly in excess compared to the random variability in F_st_ observerd in the NS.

Based on publicly available genotype data from Perlegen/HapMap we estimated trans-haplotype frequencies for 12,875,275 combinations of 5075 autosomal NSCS and compared the distribution of the genetic distances (Wright's F_st_) (Table S3 and S4) to the distribution derived from 5075 randomly selected NS. Ascertainment bias of the SNPs present in this dataset is a known limitation for using the data for population genetic studies[16], however we aim to compare two subsets of SNPs based on a property (NSCS vs. NS) not primarily involved in the SNP discovery process. In other words, we expect that the bias is similar for both NSCS and NS, although this assumption need not be correct. However, the distribution of LD (measured as r^2^) within populations among the NSCS and NS were identical indicating that the two samples are well matched with respect to potential sources of bias due to genomic localization, allele frequency spectrum and also ascertainment (Figure S1). NSCS trans-haplotypes differ significantly from those obtained from NS (Kolmogorov –Smirnof Z test, p<10^−13^) (Figure 3). The analysis was also performed with the minor haplotype frequency (MHF) as a covariate to effectively compare F_st_ values at the same level of total genetic variance. The effect of the MHF was indeed significant (p<10^−13^), but the difference between NS and NSCS based haplotypes remained highly significant as well (p<10^−13^). This result is underlined by simple visual inspection of the distributions, which reveals a much “thicker” right tail of the NSCS distribution. Although high levels of genetic differentiation were also observed among the NS, starting from approximately F_st_>0.35, two third of the data were NSCS. Hence, in this dataset the prediction that a SNP pair is NSCS rather than NS based on high levels of F_st_ alone yields a False Discovery Rate of ∼0.33. In other words, the potentially functional NSCS pairs are 2 fold overrepresented at high levels of genetic differentiation.

### Coalescent simulation of three major human populations

**Figure 4:**
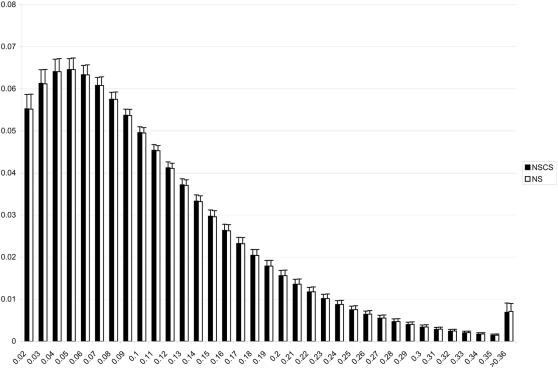
Distribution of two-locus F_st_ from a coalescent simulation of three major human populations. Simulated SNPs randomly called NSCS vs. NS exhibit no difference in pair-wise genetic distance between populations. Simulated SNPs were ascertained for being polymorphic in all three populations. Results from 100 replicates and a total of 2.5 billion combinations are summarized, with the error bars representing standard deviations.

Because both the NSCS and NS have been included randomly with respect to chromosomal position and are scattered across the entire genome, any demographic event is expected to have had the same impact on both classes of polymorphisms. Therefore, it is not likely that the clear overrepresentation of NSCS trans-haplotypes among the most strongly diverged combinations is the result of historic events. However, to estimate the variability in the amount of genetic divergence between populations due to historic processes we conducted coalescent simulations implementing parameters from a recent calibration of the method shown to accurately recreate the patterns of genetic variability between the three major world populations used here [12]. 100 replicates have been simulated resulting in a grand total of more than 2.5 billion haplotype based F_st_ values. The observed variability between replicates is very low and the combinations randomly declared NSCS vs. NS show no significant difference in the pattern of genetic differentiation (Figure 4).

### Interaction between two GABA related genes explain phenotypic differences in depression/anxiety between children

Given our interpretation that NSCS trans-haplotypes that show excess genetic differentiation between populations represent functional interactions between genes again we scrutinized our results to see whether genes known to have functional interactions from molecular studies had been identified. We prioritized the NSCS based on the number of pairs each individual SNP was involved in at Fst>0.35. This rather arbitrary cutoff was chosen because at this level of genetic differentiation the NSCS were 2 fold overrepresented compared to the NS in this dataset. Indeed, one of the NSCS (rs902790) with the highest number of reasonably strong interactions (r^2^>0.35), identified GPR156, a GABA(B) related G-protein coupled receptor. We examined all interactors of GPR156 detected here and found GABRR3 (represented by rs832032), neuregulin 3 (rs17101193), DNAI2 (rs1979370) and DEF6 (rs2395617) all of which are related to GABA receptor functioning. GABRR3 is a GABA receptor. Family members of neuregulin 3, are known to affect GABA receptor expression[17], [18]. DNAI2 is a dynein polypeptide and dynein light chains have been found to interact with Gephyrin, which in turn is a mediator of the clustering of major subtypes of the GABA(A) receptor [19]. DEF6 is an upstream activator of the Rho GTPase Rac1, a regulator of GABA(A) receptor channels in rat hippocampal neurons [20].

**Figure 5:**
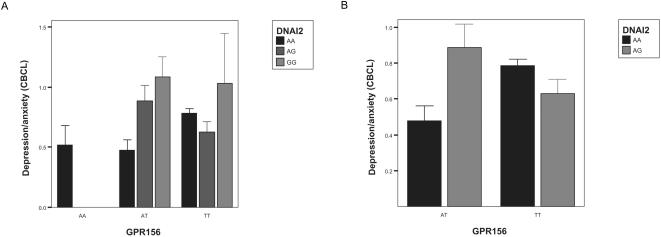
The interaction between two genes related to GABA receptor signaling is associated with anxiety/depression in a Dutch, family based cohort. A The individual effects of both GPR156 (rs902790) and DNAI2 (rs1979370) do not predict the levels of anxiety/depression in children. Instead the phenotype depends on the specific combination of protein variants present at the two genes. B Given the small number of individuals homozygous for the minor alleles at either gene (15 out of 766 individuals), only the four common genotype classes are depicted here. A significant interaction between the genes can be observed as both the double heterozygous individuals and the individuals homozygous for both major alleles exhibit increased levels of anxiety/depression. The effect size of any given protein variant is dependent on the variant present at the other locus in the same individual.

Because the GABA receptor pathway is known to be involved in depression in humans [21] we explored the possibility that individual differences in a mood related phenotype might be explained by the four combinations of the GABA related genes claimed here to be functional. The SNP rs90279 and the four NSCS ascertained for potentially functional pair-wise interactions with it were genotyped in a Dutch twin cohort that was previously phenotyped for a measure of depression/anxiety as extensively described elsewhere [22]. Following appropriate (quality) checks three pairs of protein variants were subjected to a population based association test that allows for detecting statistical interaction between the variants. No individual effect of the four NSCS analysed was significant, however the interaction between GPR156 and DNAI2 was significantly associated (p = 0.04) with childhood depression/anxiety in this cohort (Figure 5). As three tests have been performed the above finding would not be significant if Bonferroni correction would be applied, however these test are not independent as they all include rs90279. Although only marginally significant, this result is in line with the prediction that these genes are members of a co-adapted gene complex and illustrates that searching for main effects alone will could miss relevant determinants of complex phenotypes, although a replication of this association in an independent cohort would be needed before any conclusions can be drawn.

## Discussion

More than 75 years ago Sewall Wright proposed that functional interactions between genes would allow the genome to code for a virtually unlimited range of phenotypes and in fact he had produced one of the first empirical studies to show that epistastic interactions indeed are involved in determining a complex trait [23]. The existence of gene-gene interactions and their relevance for the genetics of complex traits has been generally accepted as shown by ample examples [24]–[28], but an estimate of the genome wide prevalence of epistasis and a feasible approach to ascertain functionally interacting genes from genotype data has been lacking.

A profound difficulty in a phenotype based assessment of epistasis on a genome wide scale is the need to correct for a huge number of tests with obvious consequences for statistical power. Recently strategies have been proposed to tackle this problem [29], [30], but these are yet to be put in practice. In the present approach we refrain from trying to assess the probability that an individual combination is indeed functional, instead we demonstrate an excess two-locus genetic differentiation between populations in two mammalian species. In agreement with previous results on single markers [6], [7], we observe that high levels of genetic differentiation alone is not sufficient to prove function, as both in the mouse and human analyses very high values of LD and F_st_ were indeed observed in the simulations (mice) or among the NS (humans). However, the aim of the present approach is rather to first ascertain candidate “co-adapted” genes to be tested for association with complex traits, thus strongly reducing the number of tests in the analysis of specific phenotypes. Indeed, in both the mouse and the human analysis we selected a limited subset of combinations that were likely to be involved in epistatic interactions. In both cases no main effects of these candidate “co-adapted” genes were found on the complex traits analysed. However, we observed marginally significant interaction effects of these “co-adapted” genes on ethanol consumption in mice and on anxiety/depression in humans. Replication of these associations are necessary to ascertain that they are not false positives, but the results are consistent with the prediction that the interaction between these genes rather than their individual effects should be associated with phenotypic variation.

Estimating the genome wide preference of functional interactions from these results remains difficult because divergence taking two genes into account does not necessarily have to be caused by the interaction among the genes. A strong individual effect of either of them might explain the observed pattern. While we can not exclude that strong main effects contribute to the observed patters we would argue that this can not explain the majority of the cases. If that were the case we should expect *all* combinations involving the gene/SNP with the strong main effect to yield a high signal. Clearly this is not the case demonstrated by the fact that the 707 Bonferroni significant combinations in the mouse data involved 315 SNPs. The SNP involved in the highest number of interactions was only present in 15 pairs and 40% of the 315 SNPs was involved in not more than 2 combinations. Real interactions rather than strong individual effects must clearly be involved in the majority of the cases. An obvious inference from these results is that standard genetic analyses of complex traits are bound to miss the vast majority of relevant genes. In contrast, here we show that first ascertaining candidate co-adapted genes from population genomic data followed up by an association study is a feasible way to simultaneously detect multiple susceptibility genes for complex (disease) phenotypes even if these lack any individual effect on their own.

## Materials and Methods

### LD in and simulation of mouse RILs

The genotype data on the BXD mouse recombinant inbred lines were downloaded from The GeneNetwork (http://www.genenetwork.org/dbdoc/BXDGeno.html). Linkage disequilibrium was calculated as the square of the correlation coefficient between two loci across the 89 lines and corresponding p-values were derived from a chi-squared test. Heterozygous RIL genotypes were treated as missing data. SNPs were considered unlinked if they were on different chromosomes or were more than 50 cM apart. Extreme values of allele frequencies may bias LD estimates but in RILs, allele frequencies at all loci should be by definition 0.5. In this dataset the average allele frequency was 0.4996+/−0.079 (SD). QTLCartographer was used to simulate genotyes for 89 recombinant inbred lines with 200 neutral loci per chromosome. Pairwise r^2^ values were calculated for unlinked SNPs from 1000 replicates.

### Genetic association with interactions between SNPs in mouse RILs

The phenotype data on the BXD mouse recombinant inbred lines were downloaded from Nervenet (http://www.nervenet.org/main/databases.html). For the association analysis a multiple linear regression model was implemented in R with the RIL phenotypes as dependent variables and the interaction terms of the SNP pairs as predictors. The number of BxD lines with phenotype data is much lower than the number of lines that have been genotyped, around the 20 depending on the phenotype. Because of this, in the initial screen of the 626 phenotypes only the interaction terms were considered to avoid fitting an over-parameterized model. However, the main effects were included in the analysis of the best fitting model to assess the significance of the interaction terms per se. None of the four main effects were significant in the full model which explained 66% of the phenotypic variation between the RILs. Bonferroni correction was applied to correct for the number of phenotypes tested for determining the significance of the best fitting model.

### Estimation of trans-haplotype frequencies, calculation of F_st_ between three human populations and correction for trans-haplotype frequencies and LD between markers

Genotype data for a large number of SNPs is available based on 24, 23 and 24 individuals from respectively an Asian, African-American and Caucasian population from Perlegen/HapMap. Out of the approximately 1.5 million SNPs with available genotypes, 5075 were found to be non-synonymous coding variants, polymorphic in all three populations. Unlike in mouse RILs, phase is not evident in outbred genotype data, thus trans-haplotype frequencies were estimated in each population separately with the Expectation-Maximization algorithm implemented in the LDmax software [31]. F_st_ among three populations was calculated according to the method of Weir [32], [33] that takes into account small sample estimation bias, using custom made shell scripts. Measures of genetic distance are known to be a function of the allele frequency of the loci involved. Therefore, the comparison of NS vs. NSCS distributions was repeated based on the residuals after modeling F_st_ in a linear model with all 12 trans-haplotype frequencies and 3 measures of LD (r2, one for each population) as predicting variables. Although all included predictors had a significant effect, the total amount of variance explained was small and the difference between NS and NSCS remained highly significant (Kolmogorov–Smirnof Z test, p<10^−13^).

### Coalescent simulation of three human populations

Using the cosi software for coalescent simulations we generated genotype data for 48, 46 and 48 chromosomes respectively from the three human populations. We implemented parameters as described in a recent calibration of the method [12], shown to very well approximate the real data available from the HapMap project. 22 independent autosomal chromosomes were simulated by joining the output of separate cosi runs. This way the different “chromosomes” will have different coalescent trajectories but will be subject of the same demographic history. A total of 10150 loci have been randomly generated, conditioning on all loci being polymorphic in all three populations. These 10150 loci were randomly assigned in two groups of 5075 NS and 5075 NSCS. Computational restrictions limit the size of the chromosomal fragments that can be simulated to 1.5 MB. Due to this limitation the density of the simulated polymorphisms is higher that in the real data, but the distribution of pairwise LD (r^2^) from the simulations was very similar to the real data. This could be expected as 1.5 Mb is a large enough distance for most polymorphisms to be in low LD in the general populations simulated here. Once the average distance between loci is large enough not to be in strong LD a further decrease in marker spacing is not necessary for the simulation results to be representative. The distribution of “NS” vs. “NSCS” F_st_ values were compared from this data, using 100 replicates and a total of approximately 2.5 billion genetic distances.

### Genetic association with interactions between NSCS in human family based data

Details of the Dutch twin cohort and the phenotypic measures used have been described by previously [22]. A total of 758 individuals contributed both genotype and phenotype information for this study. The cohort contained 238 monozygous and 250 dizygous twin families. For this analysis, measures of depression/anxiety obtained from children at three different ages (7, 10 and 12 years) were averaged to yield a robust estimate of the individual phenotype. Because the distribution of this measure is right skewed the data were log transformed before analysis. Genotypes for the five NSCS were determined using the ABI SNPlex Genotyping system following the manufacturer's recommendations (Applied Biosystems, Foster city, CA, USA). All pre-PCR steps were performed on a cooled block. Reactions were carried out in Gene Amp 9700 Thermocycler (Applied Biosystems, Foster city, CA, USA). PCR products were analyzed with ABI3730 Sequencer (Applied Biosystems, Foster city, CA, USA). Alleles were called using Genemapper v3.7 (Applied Biosystems, Foster city, CA, USA). Initial data analyses were performed using the pedstats and QTDT software including gender as a covariate. All NSCS were in Hardy & Weinberg equilibrium and four showed no evidence of population stratification but rs17101193 (neuregulin 3) could not be tested for population stratification due to a lack of informative families and was excluded from the population based association test. Given that no evidence for population stratification was found for the remaining four NSCS we chose to use the population based association test implemented in QTDT as it is known to have more statistical power. This is a linear model that includes variance components to model the phenotypic similarities within a family, i.e. between the twins. Standard software for family based genetic association with quantitative traits (such as QTDT) do not readily allow for testing the significance of gene-gene interactions, but QTDT does allow for testing interactions between covariates. We created dummy variables coding the three genotypes per locus −1,0 and 1 and compared the likelihood of the model including the interaction term between two loci with the likelihood of the model with only the two main effects present. The significance of the interaction term can be determined by considering minus twice the difference between the loglikelihoods of the models. This quantity has a chi squared distribution with one degree of freedom.

## Supporting Information

Table S1 (46.60 MB ZIP)

Table S2 (0.02 MB ZIP)

Table S3 (78.31 MB ZIP)

Table S4 (0.04 MB ZIP)

Figure S1 (0.73 MB TIF)
